# Translation, cross-cultural adaptation, and validation of the Los Angeles Prehospital Stroke Screen for use in Brazil

**DOI:** 10.1590/0004-282X-ANP-2020-0589

**Published:** 2022-02-28

**Authors:** Priscila Masquetto Vieira de Almeida, Rodrigo Bazan, Octávio Marques, César Minelli, José Eduardo Corrente, Gabriel Pinheiro Modolo, Gustavo José Luvizutto, Alessandro Lia Mondelli

**Affiliations:** 1 Universidade Estadual Paulista, Faculdade de Medicina de Botucatu, Botucatu, SP, Brazil. Universidade Estadual Paulista Faculdade de Medicina de Botucatu Botucatu SP Brazil; 2 Universidade de São Paulo, Faculdade de Medicina de Ribeirão Preto, Ribeirão Preto, SP, Brazil. Universidade de São Paulo Faculdade de Medicina de Ribeirão Preto Ribeirão Preto SP Brazil; 3 Hospital Carlos Fernando Malzoni, Departamento de Neurologia, Matão SP, Brazil. Hospital Carlos Fernando Malzoni Departamento de Neurologia Matão SP Brazil; 4 Universidade Federal do Triângulo Mineiro, Uberaba MG, Brazil. Universidade Federal do Triângulo Mineiro Uberaba MG Brazil

**Keywords:** Stroke, Emergency Medical Services, Early Diagnosis., Acidente Vascular Cerebral, Serviços Médicos de Emergência, Diagnóstico Precoce.

## Abstract

**Background:**

Stroke is one of the leading causes of death and neurological disability in the world. Several scales help professionals in the early recognition of the disease. However, none of these were developed in Brazil.

**Objectives:**

To translate the Los Angeles Prehospital Stroke Screen (LAPSS) into Brazilian Portuguese, and cross-culturally adapt and validate the scale in a representative sample of the Brazilian population.

**Methods:**

This study was carried out in two phases: the first consisted in the translation and cross-cultural validation of the LAPSS, and the second in a cross-sectional study with prospectively collected data in patients with suspected stroke treated in a Brazilian prehospital and referred to a stroke center. Statistical analysis was used to assess the sensitivity, specificity, and accuracy of the scale. Cohen's Kappa coefficient (κ) was used for psychometric assessment.

**Results:**

After translation and cross-cultural adaptation**,** the scale was applied to 86 patients. The scale presented a sensitivity of 83.8%, positive predictive value of 79.50%, specificity of 40.70%, negative predictive value of 47.80%, and accuracy of 77%. Cohen’s kappa coefficient was calculated using data from 26 (30.23%) patients and the results showed excellent inter-rater reliability in the majority of the items (52.96%).

**Conclusions:**

The scale was translated and cross-culturally adapted for use in Brazil. The scale presented high sensitivity and accuracy but low specificity, and the Cohen’s kappa demonstrated inter-rater reliability. The greatest difficulties occurred when the evaluation included subjective identifications. The scale excluded patients < 45 years old as stroke suspects.

## RESUMO

**Antecedentes:**

O acidente vascular cerebral (AVC) é uma das principais causas de morte e sequelas neurológicas no mundo. Muitas escalas auxiliam os profissionais no reconhecimento precoce da doença. Entretanto, nenhum desses instrumentos foi desenvolvido no Brasil.

**Objetivos:**

Traduzir a escala Los Angeles Prehospital Stroke Screen (LAPSS) para o português brasileiro, adaptar transculturalmente e validá-la na população brasileira.

**Métodos:**

Este estudo ocorreu em duas fases sendo a primeira constituída no processo de validação e adaptação transcultural da LAPSS e a segunda num estudo transversal de dados coletados prospectivamente em pacientes com suspeita de AVC tratados em um pré-hospitalar brasileiro e encaminhados a um centro de AVC. Utilizou-se análise estatística para avaliar a sensibilidade, especificidade e precisão da escala. O coeficiente Kappa de Cohen (κ) foi usado para avaliação psicométrica.

**Resultados:**

Após a tradução e adaptação transcultural, a escala foi aplicada em 86 pacientes. O instrumento final apresentou sensibilidade de 83,8%, valor preditivo positivo de 79,60%, especificidade de 40,70%, valor preditivo negativo de 47,80% e acurácia de 77%. O coeficiente Kappa de Cohen foi calculado com os dados de 26 pacientes (30,23%), demonstrando excelente confiabilidade interobservador em 52,96% dos itens da escala.

**Conclusões:**

A escala foi traduzida e adaptada transculturalmente para uso no Brasil apresentando alta sensibilidade e acurácia porém baixa especificidade. O coeficiente de Kappa demostrou confiança interobservador. A maior dificuldade ocorreu quando a avaliação física do paciente incluiu identificações subjetivas. Além disso, a escala exclui pacientes com idade menor do que 45 anos como suspeitos de AVC.

## INTRODUCTION

Brazil is currently experiencing an accelerated demographic transition process with a significant increase in the aging population. In recent decades, mortality from infectious diseases has decreased and from chronic diseases has increased significantly. Stroke is one of the main causes of mortality in the country and should be considered a medical priority^1^^,^^2^. Prehospital care is essential to improve acute interventions and reduce stroke mortality^3^^-^^5^.

The prehospital care service established in Brazil is called SAMU 192. Several studies have shown that most of the cases attended by SAMU 192 in Brazil are clinical emergencies, including cases of suspected stroke^6^^-^^9^. SAMU 192 is an important service in the acute phase of stroke because of the presence of qualified professionals who assess the patient, stabilize their health condition, and refer them to the best hospital according to the urgency. However, correct identification of stroke symptoms is not easy as they also occur in other diseases of the cardiovascular system, often leading the hospital to classify a patient as a case of suspected stroke. The proportion of strokes correctly identified by prehospital care professionals ranges from 30% to 83%^10^^-^^12^.

Although two-thirds of stroke cases occur in less developed countries, most evaluation tools have been developed in English within developed countries^13^. In the United States of America (USA), it is common to use the Los Angeles Prehospital Stroke Screen (LAPSS), which has higher sensitivity and specificity in diagnosing stroke compared to other scales. Although the LAPSS has not yet been validated in Brazil, it can contribute to the identification of stroke by prehospital care services. For this reason, this study aimed to translate the LAPSS into Brazilian Portuguese, and to cross-culturally adapt and validate it in a representative sample of the Brazilian population.

## METHODS

**Figure 1.**
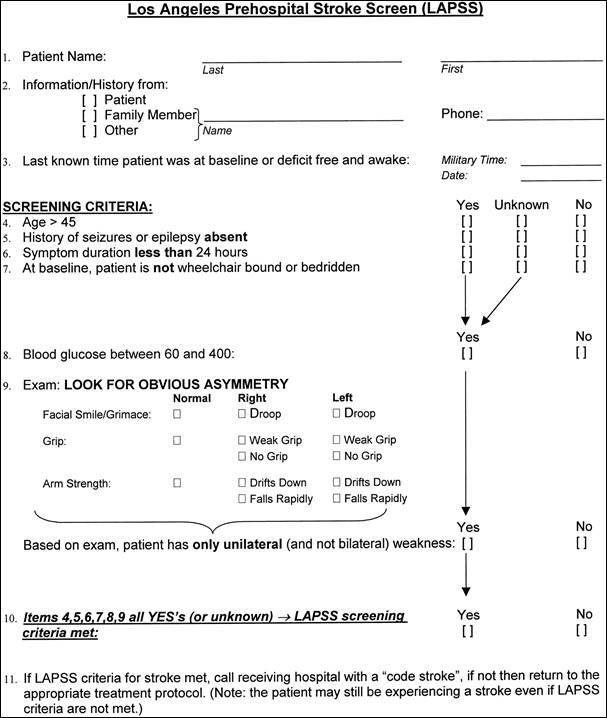
Los Angeles Prehospital Stroke Screen (LAPSS)

Source: Kidwell et al., 2000^14^.

This study was carried out in two phases: the first consisted of a systematic process of translation and cross-cultural adaptation of the original scale (Figure 1), and the second consisted of the validation of the version adapted to the Brazilian Portuguese using Cohen's kappa coefficient (κ) for psychometric assessment. 

The LAPSS was developed primarily for prehospital care professionals and consists of four items that assess the patient's history and blood glucose measurement, and three items that assess the unilateral loss of motor power. These items help to identify strokes and may also help in the exclusion of stroke mimics, a terminology used internationally to classify patients with symptoms that mimic stroke^14^^,^^15^.

The study was conducted in a city located in the interior of the state of São Paulo, Brazil, at a stroke center certified by the Brazilian Ministry of Health and a well-structured prehospital service with nationally recognized quality, named “SAMU 192”; both are part of the stroke care policy in Brazil and work in an integrated way.

SAMU 192 teams are divided into basic life support teams (nursing technicians and ambulance drivers) and advanced life support teams (doctors, nurses, and ambulance drivers). The in-hospital team consists of a multi-professional group including stroke neurologists, nurses, physiotherapists, speech therapists, occupational therapists, nutritionists, and social workers.

### Translation and cross-cultural adaptation

The first phase of the study occurred between January and June 2016 and involved ten professionals who participated directly in the process of translation and cross-cultural adaptation of the original scale^14^ according to the methodology described by Beaton et al.^13^ after prior authorization by the authors of the original scale (Supplemental material**)**.

- I. Initial translation: Two bilingual translators and native Brazilian Portuguese speakers (T1 and T2), one who was a specialist in the area and the other without knowledge on the subject, translated the original scale from English.
- II. Synthesis of translations: After the initial translations, an analysis of the divergence between evaluators was conducted and a new version (T12) of the translated scale was developed by a committee of experts. The main researcher was the mediator.
- III. Back translation: To verify that the T12 version reflected the same meaning as the original scale, two translations were performed for the original language that generated two versions (BT1 and BT2). The process was the same as in phase I, but with different bilingual translators unfamiliar with the original scale.
- IV. Analysis of the committee of experts: After the back-translation, the versions were analyzed by a committee of experts for semantic, idiomatic, conceptual, and content equivalences. This committee consisted of researchers and health professionals, in addition to the translators involved in the previous stages, and consolidated all versions of the scale (that is, T1, T2, T12, BT1, and BT2).
- V. Test of the prefinal version: The researcher interviewed the contributing professionals regarding possible doubts about the meaning of each item.

### Validation of the prefinal version

The final translated and adapted scale was applied by a SAMU 192 care team at the beginning of the assistance in cases with suspected stroke reported by the call center. The professionals were trained to use the scale, which was later applied between July 2016 and December 2017. Patients > 18 years of age, suspected of having a stroke and accompanied by a person who consented to the use of the patient data for research purposes were included in this study. All participants provided written consent, including the professionals who applied the scale and the patients. If the patient was unable to give consent, it was obtained from their legal guardian. 

Each professional applied one scale per patient. In cases of advanced life support, where there were two professionals (one doctor and one nurse), both evaluated the patient and applied the scale independently, generating two scales per patient. 

### Statistical methods 

To verify the reliability of the scale, Cohen's kappa coefficient (κ) was calculated considering < 0.20 as a poor, 0.20-0.39 as fair, 0.40-0.59 as moderate, 0.60-0.79 as good, and > 0.8 as an excellent association^16^^,^^17^. For this analysis, when the scale was applied concurrently by two professionals on the same patient, both versions were considered, which was possible in 26 cases (30.23%). 

To assess validity of the scale, sensitivity, specificity, accuracy, positive predictive value (PPV), and negative predictive value (NPV) tests were calculated, considering a 95% confidence interval. The final diagnosis of the patient was considered the "gold standard" for statistical analysis. It was performed in the hospital by a medical specialist based on the physical examination and specific imaging exams according to the internal protocol of the hospital, consisting of computed tomography and angiotomography on arrival for all patients, control computed tomography 24-48 hours from arrival, and perfusion computed tomography for patients presenting with symptoms for up to 8 hours. Patients who were not diagnosed with stroke were categorized as stroke mimics. For all statistical analyses, we used SAS version 9.4 software for Windows.

### Ethical statement

Ethics approval was granted by the Botucatu Medical School Research Ethics Committee (Protocol: 1.874.731).

## RESULTS

In the first step of this study, translation and cross-cultural adaptation of the original scale was performed. This process aimed to guarantee the semantic, idiomatic, and conceptual equivalence of the final scale (Table 1).

**Table 1.** Process of translation and cross-cultural adaptation of the Los Angeles Prehospital Stroke Screen for use in Brazil (LAPSS).

LAPSS	Final version
Los Angeles prehospital stroke screen	Escala de avaliação pré-hospitalar do AVC -LAPSS
Screening criteria	Critérios de triagem
Age over 45 years	Idade acima de 45 anos
History of seizure absent	Ausência de história prévia de crise convulsiva
New onset of neurologic symptoms in last 24 hours	Início dos sintomas neurológicos nas últimas 24 horas
At baseline, patient is not wheelchair bound or bedridden	Paciente capaz de deambular antes do quadro clínico atual
Blood glucose between 60 and 400	Glicose sanguínea entre 60 e 400
Exam:	Exame
Look for obvious asymmetry	Procure por assimetrias obvias
Normal	Normal
Right	Direita
Left	Esquerda
Facial smile / grimace	Sorriso/careta facial
Droop	Assimetria
Grip	Aperto com a mão
Weak grip	Aperto fraco
No grip	Nenhum aperto
Arm strength	Força no braço
Drifts down	Cai lentamente
Falls rapidly	Cai rapidamente
Based on exam, patient has only unilateral (and not bilateral) Weakness	Baseado no exame, paciente tem fraqueza unilateral?
If yes (or unknown) to all items above LAPSS screening criteria met	Se sim (ou desconhecido) para todos os itens acima, considerar preenchidos os critérios de triagem.
If LAPSS criteria for stroke met, call receiving hospital with "CODE STROKE", if not then return to the appropriate treatment protocol.	Se os critérios de triagem LAPSS forem preenchidos, ligue para o hospital de referência e ative o CODIGO AVC, se não, retornar para o protocolo de tratamento apropriado.
Note: the patient may still be experiencing a stroke even if LAPSS criteria are not met	Nota: o paciente pode estar apresentando um AVC mesmo se o critério LAPSS não foi encontrado.

LAPSS: Los Angeles Prehospital Stroke Screen; AVC: Acidente Vascular Cerebral.

 After this step, the final scale was applied to the target population, consisting of 86 patients with suspected stroke. Of these, 60 (69.77%) were diagnosed with stroke: 48 (80%) with ischemic stroke, 9 (15%) with hemorrhagic stroke, and 3 (5%) with transient ischemic attack. 

Cohen’s kappa coefficient was calculated for the 26 cases in which the scale was applied twice. The analysis of the psychometric characteristics of the final scale demonstrated inter-rater reliability, evidenced by the high value of Cohen’s kappa coefficient in most items. 

Table 2 shows that 52.95% of the items presented excellent inter-rater reliability among the observers and none showed poor or fair reliability. 

**Table 2.** Cohen’s kappa coefficients of the final version of the Los Angeles Prehospital Stroke Screen translated and adapted for use in Brazil.

Scale items	κ	95% CI
History of seizure absent	0.8354	0.5235 -1.0000
At baseline, patient is not wheelchair bound or bedridden	1.0000	1.0000 -1.0000
Facial smile / grimace (normal)	0.8976	0.7019 -1.0000
Facial smile / grimace (right)	0.7541	0.4941 -1.0000
Facial smile / grimace (left)	0.6601	0.3557 -0.9645
Grip (normal)	0.9128	0.7457 -1.0000
Weak grip (right)	0.6929	0.3717 -1.0000
No grip (right)	0.8207	0.5858 -1.0000
Weak grip (left)	0.5979	0.1891 -1.0000
No grip (left)	0.7797	0.3666 -1.0000
Arm strength (normal)	0.9128	0.7457 -1.0000
Arm strength / drifts down (right)	0.7079	0.3358 -1.0000
Arm strength / falls rapidly (right)	0.9128	0.7457 -1.0000
Arm strength / drifts down (left)	0.5063	0.0241 -0.9886
Arm strength / falls rapidly (left)	0.8354	0.5235 -1.0000
Based on exam, patient has only unilateral (and not bilateral) weakness	0.6929	0.3717 -1.0000
If yes (or unknown) to all items above LAPSS screening criteria met	0.8308	0.5114 -1.0000

κ: Cohen’s kappa coeficiente; CI: confidential interval = 95%; LAPSS: Los Angeles Prehospital Stroke Screen.

Statistical analysis revealed a sensitivity of 83.8% (95% CI [75.40 -92.19%]; NPV of 47.80%), specificity of 40.70% (95% CI [22.17 -59.25%]; PPV of 79.50%), and accuracy of 77% (95% CI [68.79 -82.21%]).

## DISCUSSION

In this study, we translated and performed a cross-cultural adaptation of the LAPSS for use in Brazil. Furthermore, we applied the final scale into a sample target population and analyzed the obtained results. The scale presented values for sensitivity, accuracy, and inter-rater-reliability as in other similar studies^18^. There was divergence in the application of the scale between evaluators in some subjective items showing moderate agreement. 

 The prehospital stroke scales recommended by the American Heart Association and European Stroke Association were created in developed in countries that do not have the same health profile as the Brazilian population. In addition, some of these countries have prehospital care services with structures different than Brazil and other underdeveloped countries. A systematic review of recent literature has shown that the main scales used in prehospital care services are from validation studies that were mostly performed in developed countries^18^.

In this way, it is fundamental that these scales be systematically translated and adapted to the local population. Often, these scales undergo a simple translation and are implanted in the service routines, which does not ensure the semantic, idiomatic, experiential, conceptual, and content equivalence of the final scale. In this study, we opted for the widely used theoretical framework described by Beaton et al^13^. The study described steps to ensure translation quality and cross-cultural adaptation that aimed to keep the final scale as close as possible to the original while still making it applicable to the target population. In addition, some psychometric tests were included to analyze the validity and reliability of the scale.

The scale is divided into four different parts: the first refers to the collection of the patient's medical history, the second refers to the verification of blood glucose, the third consists of data on the current physical examination, and the fourth consists of questions to confirm all previously evaluated items^14^.

In the first part, there are four items related to the patient's medical history: age > 45 years old, history of seizures, onset of neurological symptoms in the last 24 hours, and ability to ambulate. These items help professionals identify stroke and make decisions regarding the referral priority of patients. In this study, inter-rater reliability was excellent, possibly because of the objectivity of the information, which was asked to the patient’s family or companion. According to a previous study, patients aged < 45 years usually present with acute weakness associated with other etiologies, and postictal patients also present with transient paralysis^14^. However, there is evidence of an increase in stroke cases in young patients aged < 45 years, especially in Latin American countries, including Brazil, where studies show a high risk of stroke in patients aged 40 to 79 years and a high incidence in the population < 40 years^19^^,^^20^. Based on this, it is considered that this item may impair the identification of stroke in young patients.

The verification of blood glucose in the second part of the LAPSS is aimed to exclude the possibility of hypoglycemia, which may also present symptoms similar to a stroke. Hypoglycemia is a common condition in patients presenting in the prehospital care^6^, which makes its identification crucial before the patient is referred to a stroke center. As this was a one-time procedure during service and its result was shared among the team, there was no divergence in the score of this item.

In the third part, the scale presents a physical examination script to identify unilateral motor deficit. The script helps professionals exclude patients with non-focal weakness due to intoxication or systemic disease, among other conditions. The results show that the professionals presented less divergence when the evaluated item was normal, showing an excellent inter-rater-reliability. However, there was a greater divergence in the identification of altered items, especially when the evaluation included subjective identifications such as "weak grip" and " drifts down". Despite this, there was a moderate inter-rater-reliability for these two items, which did not significantly interfere with the reliability of the scale, since the professionals identified the unilateral weakness, regardless of the degree. However, identifying the unilaterally weakness can be difficult, which is done, for example, by analyzing the item “weak grip”, a subjective item in this exam. We believe that stroke assessment scales should be used as objectively as possible to better identify suspected cases and consequently provide better patient care. Given the characteristics of Brazilian prehospital care, where most professionals are not physicians, the subjectivity of the physical examination script can significantly interfere with the final result of the scale, increasing the number of false negatives. 

In the fourth part, the last questions showed different levels of agreements. There was strong agreement in the analysis of unilateral weakness, which was expected based on the difficulty of inter-rater reliability in the physical examination. The last question, where the professionals verify if all items of the screening criteria are met, had high reliability.

The results showed values ​​for sensitivity similar to those reported in the literature, that is, the scale had similar sensitivity to its application in patients from the USA, Canada, the United Kingdom, Australia, and China. In contrast, specificity was lower than that reported by studies performed in other countries^21^.

One of the advantages of using the LAPSS is that it could help practitioners differentiate stroke cases from stroke mimics. However, the NPV of the scale was 47.80%, a low value considering that stroke is one of the main causes of mortality in the country. Because stroke is one of the main causes of mortality and physical disability in Brazil, prehospital care evaluation scales should be as sensitive as possible, regardless of the specificity of the scale. This allows a greater number of patients to have access to a stroke center where stroke mimics can be safely ruled out. This may increase the diagnosis of the disease in Brazil and, consequently, decrease the number of deaths and disability caused by late diagnosis.

In general, the LAPSS is not easy to apply. There are four parts with two response patterns, dichotomous and categorical. In addition, it is essential to think about its extensive use as an effective scale for prehospital professionals since this service needs a fast, simple, and accurate scale. This does not exclude the possibility of using this scale in emergency units, where better structural conditions exist for its application.

This study has some limitations. One limitation was the fact that the SAMU 192 team did not have a report of each patient’ specific complaints, so it was impossible to know how many cases of stroke the team treated during the period of data collection. Another limitation was based on the use of the scale, as some items are subjective and the ability to distinguish them is different for each professional. Furthermore, we were unable to assess intra-observer reliability because in prehospital care it is not possible to apply the scale in two moments by the same evaluator. Perhaps this possibility exists if the scale is applied in the hospital. 

In this study, we decided to include only a prehospital care team based on the profile of this service in Brazil. The model of prehospital care in the USA is different from that in Brazil, but no care center has expert raters. The original scale was developed to be applied by paramedics in the USA. It is important that as many professionals as possible know how to identify stroke in the field, as this will help increase treatment rates and decrease death and disability.

Further studies should be carried out with the aim of analyzing a scale more suited to the profile of the Brazilian population. A simpler, easier-to-use scale is the way to qualify prehospital care teams for early identification of stroke, which would increase referral of patients to specialized centers and decrease death and disability due to delayed care.

Although the study was carried out in a privileged location regarding the delivery of health services, the results can be disseminated and used throughout the national territory, as the terminology widely used among professionals working in the prehospital setting.

In conclusion, the scale was translated and cross-culturally adapted for use in Brazil. The scale presented high sensibility and accuracy but low specificity. The Cohen’s kappa demonstrated excellent inter-rater reliability in 52.96% of the items. The major difficulty occurred when evaluation included subjective identifications, such as weak grip and arm strength -drifts down/falls rapidly. The scale excluded patients < 45 years old as suspects of stroke. 
