# Supplementary material for: Translation, cross-cultural adaptation, and validation of the Los Angeles Prehospital Stroke Screen for use in Brazil
Source: Arq Neuropsiquiatr. 2022 Feb 28;80(3):217–23. doi: 10.1590/0004-282X-ANP-2020-0589 (PMC9648932; doi:10.1590/0004-282X-ANP-2020-0589)
Supplement: Supplementary file 1 [file 1678-4227-anp-80-03-217-s1.pdf]

https://doi.org/10.1590/0004-282X-ANP-2020-0589

SUPPLEMENTARY MATERIAL

Process of translation and cross-cultural adaptation of the Los Angeles Prehospital Stroke Screen Brazilian Portuguese language.

LAPSS Items	T1	T2	Version T 1-2	BT1	BT2	Version BT 1-2	Final version*
Los Angeles prehospital stroke screen	Triagem pré-hospitalar de acidente vascular cerebral de Los Angeles	Quadro pré-hospitalar de acidentes vasculares de Los Angeles	Triagem pré-hospitalar de acidente vascular cerebral De Los Angeles	Los Angeles prehospital stroke screen / screening / triage	Los Angeles prehospital stroke screen (LAPSS)	Triagem pré-hospitalar de acidente vascular cerebral de Los Angeles	Escala de avaliação pré-hospitalar do AVC - LAPSS
Screening criteria	Critérios de triagem	Critérios de classificação	Critérios de triagem	Screening / Triage criteria	Screening criteria	Critérios de triagem	Critérios de triagem
Age over 45 years	Idade acima de 45 anos	Idade superior a 45 anos	Idade acima de 45 anos	Age above 45 years	Over 45 years old	Idade acima de 45 anos	Idade acima de 45 anos
History of seizure absent	Ausência de história prévia de crises epilépticas	Sem histórico prévio de distúrbio epilético	Ausência de história previa de crise convulsiva	Absence of prior history of convulsive crisis	Absence of previous seizure history	Sem história prévia de crise convulsiva	Ausência de história prévia de crise convulsiva
New onset of neurologic symptoms in last 24 hours	Surgimento de sintomas neurológicos novos nas últimas 24 horas	Nova aparição de sintomas neurológicos nas últimas 24 horas	Surgimento de sintomas neurológicos novos nas últimas 24 horas	Development of new neurological symptoms in the last 24 hours	Development of new neurological symptoms in the last 24 hours	Novo início de sintomas neurológicos nas últimas 24 horas	Início dos sintomas neurológicos nas últimas 24 horas
At baseline, patient is not wheelchair bound or bedridden	O paciente não está em cadeira de rodas ou acamado	O paciente não está em cadeira de rodas ou acamado (antes do evento)	Paciente capaz de deambular antes do quadro	Patient capable of walking before the frame / clinical picture	Patient able to walk before the clinical condition	Paciente capaz de deambular antes do quadro	Paciente capaz de deambular antes do quadro clínico atual
Blood glucose between 60 and 400	Glicose sanguínea entre 60 e 400	Glicose do sangue entre 60 e 400	Glicose sanguínea entre 60 e 400	Glycemia between 60 and 400	Glycemia between 60 and 400	Glicose sanguínea entre 60 e 400	Glicose sanguínea entre 60 e 400
Exam:	Exame	Exame	Exame	Exam	Exam	Exame	Exame
Look for obvious asymmetry	Procure por assimetrias obvias	Busca por assimetria óbvia	Procure por assimetrias obvias	Look for obvious asymmetries.	Look for obvious asymmetries	Procure por assimetrias obvias	Procure por assimetrias obvias
Normal	Normal	Normal	Normal	Normal	Normal	Normal	Normal
Right	À direita	Direita	À direita	To the right	On the right	Direita	Direita
Left	À esquerda	Esquerda	À esquerda	To the left	On the left	Esquerda	Esquerda
Facial smile / grimace	Mimica facial ao sorriso	Sorriso/careta facial	Mimica facial ao sorriso	Facially mimics a smile	Facial mime while smiling	Sorriso/careta facial	Sorriso/careta facial
Droop	Apagamento de um lado	Inclinação	Apagamento de um lado da face	Inactivity / Paralysis (Apagamento) on one side of the face	Effacement of one side of the face	Inclinação de um lado	Assimetria
Grip	Apertar com a mão	Aperto de mão	Apertar com a mão	Gripping with the hand	Grip with the hand	Aperto com a mão	Aperto com a mão
Weak grip	Fraqueza ao apertar com a mão	Aperto fraco	Fraqueza ao apertar com a mão	Weakness upon gripping with the hand	Weakness when gripping with the hand	Aperto fraco	Aperto fraco
No grip	Incapaz de apertar com a mão	Sem aperto	Incapaz de apertar com a mão	Incapable of gripping with the hand	Unable to grip with the hand	Nenhum aperto	Nenhum aperto
Arm strength	Força no braço	Força do braço	Força no braço	Weakness in the arm	Weakness in the arm	Força no braço	Força no braço

LAPSS Items	T1	T2	Version T 1-2	BT1	BT2	Version BT 1-2	Final version*
Drifts down	Braço cai lentamente	Movimenta para baixo	Braço cai lentamente	Arm falls slowly	Arm falls slowly	Movimenta lentamente para baixo	Cai lentamente
Falls rapidly	Braço cai rapidamente/ despenca	Cai rapidamente	Braço cai rapidamente/ despenca	Arm falls rapidly/ plummets	Arm falls quickly/drops	Cai rapidamente	Cai rapidamente
Based on exam, patient has only unilateral (and not bilateral) Weakness	Baseado no exame, paciente tem fraqueza somente unilateral (e não bilateral).	Baseado no exame, paciente tem somente fraqueza unilateral (e não bilateral).	Baseado no exame, paciente tem fraqueza somente unilateral (e não bilateral).	Based on the exam, the patient has only unilateral weakness (and not bilateral).	Based on the examination, the patient only has unilateral weakness (not bilateral).	Baseado no exame, paciente tem fraqueza somente unilateral (e não bilateral)	Baseado no exame, paciente tem fraqueza unilateral?
If yes (or unknown) to all items above LAPSS screening criteria met	Se sim (ou desconhecido) para todos os itens acima, considerar preenchidos os critérios de triagem.	Se sim (ou desconhecida) para todos os itens acima, classificação de critério LAPSS encontrada.	Se sim (ou desconhecido) para todos os itens acima, considerar preenchidos os critérios de triagem.	If the response is "yes" (or "don't know") to all the above items, consider the screening / triage criteria fulfilled	If true (or unknown) for all of the above, consider the screening criteria fulfilled	Se sim (ou desconhecido) para todos os itens acima, considerar preenchidos os critérios de triagem.	Se sim (ou desconhecido) para todos os itens acima, considerar preenchidos os critérios de triagem.
If LAPSS criteria for stroke met, call receiving hospital with "CODE STROKE", if not then return to the appropriate treatment protocol.	Se os critérios de triagem LAPSS forem preenchidos, ligue para o hospital de referência e ative o CODIGO AVC, se não, retornar para o protocolo de tratamento apropriado.	Se o critério LAPSS para acidentes vasculares foi detectado, ligar para o hospital receptor com "CÓDIGO AVC", se não, então retornar ao protocolo de tratamento apropriado.	Se os critérios de triagem LAPSS forem preenchidos, ligue para o hospital de referência e ative o CODIGO AVC, se não, retornar para o protocolo de tratamento apropriado.	If the LAPSS screening / triage criteria were met, call the referring hospital and activate the STROKE / CVA CODE; if not, return to the appropriate treatment protocol.	If LAPSS screening criteria are met, call the hospital of reference and activate the CODE STROKE, if not, return to the appropriate treatment protocol.	Se os critérios de triagem LAPSS forem preenchidos, ligue para o hospital de referência e ative o CODIGO AVC, se não, retornar para o protocolo de tratamento apropriado.	Se os critérios de triagem LAPSS forem preenchidos, ligue para o hospital de referência e ative o CODIGO AVC, se não, retornar para o protocolo de tratamento apropriado.
Note: the patient may still be experiencing a stroke even if LAPSS criteria are not met	Nota: o paciente pode estar tendo um AVC, mesmo se os critérios de LAPSS não forem preenchidos.	Nota: o paciente pode ainda estar passando por um AVC mesmo que o critério LAPSS não foi encontrado.	Nota: o paciente pode estar tendo um AVC, mesmo se os critérios de LAPSS não forem preenchidos.	Note: the patient may be having a stroke, even if the LAPSS criteria had not been met.	Note: the patient may be having a stroke, even if the LAPSS criteria are not met.	Nota: o paciente pode ainda estar passando por um AVC mesmo que o critério LAPSS não foi encontrado.	Nota: o paciente pode estar apresentando um AVC mesmo se o critério LAPSS não foi encontrado.
